# Plasma concentrations of 25-hydroxyvitamin D among Jordanians: Effect of biological and habitual factors on vitamin D status

**DOI:** 10.1186/1472-6890-11-8

**Published:** 2011-08-04

**Authors:** Eyad M Mallah, Mohammad F Hamad, Mays A ElManaseer, Nidal A Qinna, Nasir M Idkaidek, Tawfiq A Arafat, Khalid Z Matalka

**Affiliations:** 1 Department of Pharmaceutical Medicinal Chemistry and Pharmacognosy, Faculty of Pharmacy and Medical Sciences, Petra University, Amman, Jordan; 2 Department of Pharmacology and Biomedical Sciences, Faculty of Pharmacy and Medical Sciences, Petra University, Amman, Jordan; 3 Department of Pharmaceutics and Pharmaceutical Technology, Faculty of Pharmacy and Medical Sciences, Petra University, Amman, Jordan

**Keywords:** (25-OHD), Vitamin D_2_, Vitamin D_3_, Diet, Dress Styles, Hypovitaminosis D, Jordan

## Abstract

**Background:**

Vitamin D is cutaneously synthesized following sun exposure (vitamin D_3_) as well as it is derived from dietary intake (vitamin D_3 _and D_2_). Vitamin D_2 _and D_3 _are metabolized in the liver to 25-hydroxyvitamin D (25(OH)D). This metabolite is considered the functional indicator of vitamin D stores in humans. Since Jordan latitude is 31°N, cutaneous synthesis of vitamin D_3 _should be sufficient all year round. However, many indications reveal that it is not the case. Thus, this study was conducted to determine the 25(OH)D status among Jordanians.

**Methods:**

Three hundred healthy volunteers were enrolled in a cross sectional study; 201 females and 99 males. 25(OH)D and calcium concentrations were measured by enzyme linked immunosorbent assay and spectroscopy techniques, respectively. All participants filled a study questionnaire that covered age, sex, height, weight, diet, and dress style for females. Females were divided according to their dress style: Western style, Hijab (all body parts are covered except the face and hands), and Niqab (all body parts are covered including face and hands).

**Results:**

The average plasma 25(OH)D levels in males and females were 44.5 ± 10.0 nmol/l and 31.1 ± 12.0 nmol/l, respectively. However, when female 25(OH)D levels were categorized according to dress styles, the averages became 40.3, 31.3 and 28.5 nmol/l for the Western style, Hijab and Niqab groups, respectively. These 25(OH)D levels were significantly less than those of males (p < 0.05, 0.001, 0.001, respectively). In addition, the plasma 25(OH)D levels of the Western style group was significantly higher than those of Hijab and Niqab groups (p < 0.001). Furthermore, dairy consumption in males was a positive significant factor in vitamin D status. Even though calcium concentrations were within the reference range, the Hijab and Niqab-dressed females have significantly less plasma calcium levels than males (p < 0.01).

**Conclusions:**

Very low plasma 25(OH)D levels in females wearing Hijab or Niqab are highly attributed to low sunlight or UVB exposure. In addition, most of males (76%) and Western style dressed females (90%) have 25(OH)D concentrations below the international recommended values (50 nmol/l), suggesting that although sun exposure should be enough, other factors do play a role in these low concentrations. These findings emphasize the importance of vitamin D supplementation especially among conservatively dressed females, and determining if single nucleotide polymorphisms of the genes involved in vitamin D metabolism do exist among Jordanians.

## Background

Vitamin D is obtained from two sources: the action of sunlight on the skin (Vitamin D_3_) and diet (Vitamin D_3 _and D_2_) [1,2]. Because few types of food provide a natural source of vitamin D, skin synthesis is thought to constitute the major source [1,2]. Vitamin D is a generic term referring to Vitamin D_3 _(cholecalciferol) and Vitamin D_2 _(ergocalciferol) where both are metabolized in an identical manner [3]. Vitamin D is readily metabolized in the liver to 25-hydroxyvitamin D (25(OH)D) which is the most abundant form of vitamin D in circulation [4]. Further metabolism of 25(OH)D to the active metabolite 1,25-dihydroxyvitamin D occurs in the kidneys [5]. Although 1,25(OH)D is the biologically active form of vitamin D, it is not the ideal measure for vitamin D status. The main reasons for that is that the plasma half life of circulating 1,25(OH)D is only 4-6 hours and the circulating levels of 1,25(OH)D are thousand fold less than 25(OH)D [6].

25(OH)D is the major circulating form of vitamin D that has a half life of approximately 2-3 weeks [6,7]. It is used to determine whether a patient is vitamin D deficient, sufficient or intoxicated [6-9]. Normal levels of 25(OH)D is necessary to maintain regular bone metabolism and bone growth [1,6-12]. The chronic and severe vitamin D deficiency leads to depletion of bone reservoirs of calcium and phosphate and insufficient bone matrix mineralization, which is a risk for developing rickets in children and osteomalacia in adults [1,6-12]. Low levels of vitamin D metabolites are associated with malabsorption of calcium, which results in bone loss [9-12]. Moreover, prostate, colon and breast cancers, hypertension, lack of proper immune modulation and diabetes are among other conditions which are related to vitamin D deficiency [12-14].

Sunlight plays an essential role in vitamin D status and since Jordan's latitude is 31°N, sun or UVB radiation exposure should be sufficient for Vitamin D_3 _synthesis, and thus 25(OH)D, all year [15]. However, studies performed on populations living on similar latitudes showed deficiency in vitamin D [16-19]. The aim of the present study is to determine the prevalence of vitamin D deficiency by measuring plasma levels of 25(OH)D, and to study the biological and habitual factors that might be positive predictors of vitamin D deficiency among apparently healthy people living in Jordan. This study and others should help in performing a strategy to combat vitamin D deficiency in such part of the world in order to reduce musculoskeletal and other metabolic diseases associated with such deficiency.

## Methods

### Study population

This study was performed in November 2010 to determine the prevalence of vitamin D deficiency in apparently healthy Jordanian volunteers. The study has been approved by the Faculty of Pharmacy and Medical Sciences and the Deanship of Scientific Research at Petra University and was performed in compliance with the Helsinki Declaration following the approval of an independent ethics committee at Al-Mowasah Hospital in Amman, Jordan. After obtaining an informed consent from 300 healthy volunteers, study questionnaire was filled for all participants. This questionnaire included age, gender, weight, height, family history of bone fracture, frequency of diet, different dress styles for women, and multi-vitamins supplementation.

A total of 201 females and 99 males were enrolled in this study. Females were divided according to their dress style: Western, Hijab (all body parts are covered except the face and hands), and Niqab (all body parts are covered including face and hands). Volunteers were also divided into their frequency of type food consumption per week as 1 (low), 3 (medium), or 6 (high) and averaged as in Table 1.

**Table 1:** Characteristics of the volunteers in each category

Parameter					p value
**Sex**	**Males**	**Females**			
	33.7%	66.3%			p < 0.001

Dress Style (Females)		Western-Style	Hijab	Niqab	
		13.3%	46.7%	7.3%	p < 0.001

Age	29.0 ± 9.7	22.5 ± 5.2*	33.9 ± 14.9†	39.3 ± 15.3†	p < 0.01

BMI	25.5 ± 3.5	21.3 ± 3.0*	24.9 ± 4.5	27.1 ± 4.6	p < 0.001

Type of Food consumption per week					
*Dairy†† *	4.0 ± 1.8*	3.9 ± 2.0	4.6 ± 1.8	5.0 ± 1.6	p < 0.01
*Meat*	3.4 ± 1.9*	4.3 ± 1.8	3.9 ± 1.8	4.1 ± 1.6	p < 0.05
*Vegetarian*	5.1 ± 1.6	4.6 ± 1.8	5.2 ± 1.5	5.5 ± 2.0	n.s
*Fish*	1.2 ± 0.7	1.7 ± 1.5†	1.3 ± 0.7	1.3 ± 0.7	p < 0.05

% of subjects using multivitamins supplement	9%	16%	27%	36%	n.s.

* and † refer to significantly different values; *:significantly less than other groups, †: significantly higher than other groups

†† Most of the dairy products is not fortified with vitamin D

### Blood Sampling and Laboratory Analysis

Blood samples were drawn into heparinized tubes, mixed, centrifuged and plasma samples were stored frozen at -70°C until the day of analysis. Plasma 25(OH)D was assayed using an enzyme linked immunosorbent assay kit (Immunodiagnostic System Ltd., Boldon Business Park, Boldon, Tyne and Wear, UK) according to the manufacturer instructions. This method has 100% and 75% specificity for 25(OH)D_3 _and 25(OH)D_2_, respectively, and has a lower limit of detection of 2 ng/ml. The intra- and inter-assay coefficients of variation were 5.3% and 4.6% respectively. Samples were run in duplicate with quality control samples to ensure day-to-day validity of results. The absorbance values were read using a plate reader (SCO GmbH, Dingelsadt, Germany) and a standard curve was plotted using the 4-parameter logistic (4-PL) model. This function provides an accurate depiction of the sigmoidal relationship between the measured response and the analyte concentration [20].

Calcium levels were measured using a commercial assay kit (Arsenazo, Biosystem, Spain) using spectroscopy technique (Evolution 160 UV-VIS spectrophotometer, λ = 650 nm). In addition, calcium concentrations were corrected for albumin concentrations and hyperlipidemia.

### Data Analysis

Descriptive statistics methods were applied in order to estimate prevalence of vitamin D deficiency. Data were analyzed using ANOVA followed by Tukey's (95% confidence) as a post hoc test; numerical data were checked for normal distribution and described as mean and standard deviation. Interaction variables were created to assess possible effect modification between age, dress style, diet, and BMI. The significance level was set at *p *< 0.05.

## Results

Three hundred healthy volunteers were enrolled in this study to quantify plasma levels of 25(OH)D and calcium. Participants consisted of 66.3% females and 33.7% males. The females were divided according to their dress style: Hijab 46.7%, Western 13.3%, and Niqab 7.3%. The mean age was 29 and 32 years for males and females, respectively. Descriptive characteristics of these volunteers are illustrated in Table 1. The frequencies of eating dairy and meat products by males were significantly less than by females. Although, all volunteers seemed to consume very low frequency of fish products, the frequency of Western style-dressed females of eating fish products was significantly higher than the other groups (Table 1).

The average plasma 25(OH)D levels in male and females were 44.5 ± 10 nmol/l and 31.1 ± 12 nmol/l, respectively. However, when female 25(OH)D levels were categorized according to dress styles, the averages became 40.3, 31.3 and 28.5 nmol/l for the Western style, Hijab and Niqab groups, respectively (Table 2). These 25(OH)D levels were significantly less than those of males (p < 0.05, 0.001, 0.001, respectively). In addition, the plasma 25(OH)D levels of the western style group was significantly higher than those of Hijab and Niqab groups (p < 0.001) (Table 2).

**Table 2:** Plasma levels of 25(OH)D and calcium among Jordanians

Parameter	Males	Females (according to dress style)		
		Western Style	Hijab	Niqab
25(OH)D (nmol/l)	44.5 ± 10^1^	40.0 ± 8.3^2^	31.3 ± 6.3	28.5 ± 3.8

Calcium (mmol/l)	2.33 ± 0.28^3^	2.25 ± 0.25	2.23 ± 0.25	2.18 ± 0.24

^1^Value is significantly higher than female groups by (p < 0.05, 0.001 and 0.001), ^2 ^value is significantly (p < 0.001) higher than Hijab and Niqab dressed females

^3^Value is significantly (p < 0.01) higher than female groups

The frequency of dairy consumption was the only observed significant factor on 25(OH)D levels and in males group only (p < 0.01). The average plasma 25(OH)D level in males of high frequency dairy consumption was 48.1 ± 11.3 nmol/l compared to 43 ± 8.3 and 37.3 ± 7.8 nmol/l, for the middle and low frequency dairy consumption, respectively. None of the other dietary factors showed any dependency on the 25(OH)D levels in our studied males or females population.

Although the average of plasma calcium concentrations were within the reference range (2.12-2.63 mmol/l), males plasma calcium levels were significantly higher than those of Hijab and Niqab dressed females (p < 0.01). There was no difference in plasma calcium concentrations between Western style, Hijab, and Niqab dressed female groups (p > 0.05) (Table 2).

Since vitamin D deficiency and insufficiency depends on the cutoff values, the frequency and distribution of plasma 25(OH)D levels are presented in Table 3. More than 90% of our studied healthy population have 25(OH)D of < 50 nmol/l. If categorized, 76% of males plasma 25(OH)D levels were below < 50 nmol/l versus 90%, 98.6% and 100% of Western style, Hijab, and Niqab dressed female groups, respectively.

**Table 3:** Distribution of 25(OH)D levels in plasma according to gender and females dress style

Vitamin D levels	Males	Females (according to dress style)		
		Western Style	Hijab	Niqab
< 25 nmol/l	0.0%	0.0%	4.3%	8.7%

25.0 - < 37.5 nmol/l	25.2%	35.9%	88.5%	87.0%

37.5 - < 50.0 nmol/l	50.5%	53.8%	5.8%	4.3%

50.0 - < 62.5 nmol/l	18.3%	10.3%	0.7%	0.0%

62.5 - < 75.0 nmol/l	4.0%	0.0%	0.7%	0.0%

≥ 75 nmol/l	2.0%	0.0%	0.0%	0.0%

## Discussion

Since the latitude of Amman, Jordan is 31°N, cutaneous Vitamin D_3 _synthesis should be sufficient all year round [15]. However, this is not the case in Jordanian males or Western dress style females. Cutaneous Vitamin D_3 _synthesis starts with UVB (290-315 nm) exposure. UVB converts 7-dehydrocholesterol to pre-vitamin D_3 _which is then converted to vitamin D_3 _by thermal isomerization. Continued skin exposure to UVB radiation, however, can result in the breakdown of pre-vitamin D_3 _and vitamin D_3 _to inactive photoproducts [21]. This process is thought to be a regulatory process of vitamin D synthesis [22]. Therefore, excess sun exposure can lead to inactive photoproduct and a decrease in vitamin D status. In addition, levels of UVB reaching the skin site for vitamin D synthesis can be attenuated by clothing, sunscreen, or skin's melanin content [21-24]. In the present study, although Jordanian males and Western dress style females were shown to possess significantly higher vitamin D levels than those of low sun exposure females (Hijab and Niqab dressed females), nonetheless, 80% of the former groups have insufficient levels (<50 nmol/l). Therefore, several reasons other than sun exposure could be involved in such reduction of vitamin D levels.

Jordanians and Middle-Eastern in general have darker skin textures i.e reflecting higher melanin production in response to UV radiation. Melanin absorbs electromagnatic radiation and competes with 7-dehydrocholesterol for UVB photons [25]. The latter may be one of the reasons of low vitamin D status among Jordanians and other Middle-Eastern countries that are located below 35°N of the equator [26-30].

Vitamin D status is also affected by seasons especially in areas of high latitude (>40°N). It has been demonstrated that measuring 25(OH) D levels in the summer is relatively higher than winter times [21,31]. However, this is also associated with the latitude of the city or the country where the study is performed. For instance, people living at 23.5-66.5° latitude may lack sufficient UVB to synthesize vitamin D during one month of the whole year, whereas other studies reported that below 35° the UVB exposure is enough for vitamin D synthesis throughout the year [28,31]. In addition, Jordan wintertime starts mid of January [28,29]. Therefore, the timing of the current study should not be the reason of the low levels seen for 25(OH) D.

Since clothing is a major blocker to sun exposure and thus vitamin D synthesis and status, this study has shown that females dressing Hijab (uncovered face and hands) or Niqab (i.e. covering all their bodies) have less 25(OH)D plasma levels than their counterparts Western style-dressed females living in Jordan. In addition, sun exposure to uncovered face and hands as in Hijab- dressed females is not sufficient for vitamin D synthesis. It has to be mentioned that the texture of clothing such as non-synthetic fibers (cotton or lenin) are less effective in blocking UV radiation than wool, silk, nylon and polyester. In addition, a lighter color of cotton clothing such as white is less effective in blocking ultraviolet light than black [23,24]. These factors might contribute to some variations herein; however, such clothing factors were not addressed in the study.

The present study supports the fact that the frequency of dairy consumption is a good source of vitamin D. Although males in Jordan consumes less dairy products than females, their 25(OH)D levels were significantly dependent on dairy consumption. Thus, it is concluded that sun exposure and dairy consumption are the main factors that could keep vitamin D levels close to the recommended values as much as possible.

Vitamin D (D_2 _or D_3_) is absorbed in the gastrointestinal tract, binds to chylomicrons, and is transported via lymphatic system to blood circulation. Circulating vitamin D, whether from diet or sun exposure, enters the circulation, binds to vitamin D-binding protein, and transported to liver for metabolism or adipose tissue for storage. In the liver vitamin D is metabolized to 25(OH)D by the vitamin D hydoxylase and 25(OH)D which is further modified to the active form 1,25(OH)2D in the kidneys. The rate and extent of the elevation of plasma 25(OH)D levels are dependent on the vitamin D hydroxylase. In addition, 25(OH)D goes through inactivation by cytochromes P-450 CYP24 (or 25(OH)D-24-hydroxylase) and CYP3A4. In addition, vitamin D-binding protein (group-specific component; GC) is also cofactor in the plasma levels of 25-(OH)D. Therefore, single nucleotide polymorphism (SNP) markers in the genes, namely CYP2R1 and GC, might contribute to the variations of 25(OH)D levels in healthy Caucasians [32,33]. Therefore, it is better that optimal concentrations of 25(OH)D in different populations should be defined in order to reduce certain diseases due to high vitamin D. Further research is required in order to clarify the genetic architecture underlying 25(OH)D plasma concentrations among Jordanians.

Plasma calcium concentration is regulated by 1,25(OH)D and parathyroid hormone (PTH). 1,25(OH)D increases calcium absorption from the intestines and PTH increases plasma calcium levels by inducing renal calcium reabsorption and stimulating bone resorption. Thus, it is essential to keep vitamin D levels sufficient in order to keep calcium level normal within the body and reduce PTH action on the bones. The present study demonstrates that the mean plasma calcium levels are less in the groups with less 25(OH)D levels. Therefore, it is essential to supplement females with vitamin D to reduce the process of osteoporosis as early as possible of their lives [34].

The daily needs of vitamin D is still under debate and controversial. The Institute of Medicine (IOM) published its recent report regarding the dietary reference intakes and revealed that 600 IU/day is the recommended daily allowance for individuals between 1-70 years of age [35]. These recommended daily allowances, however, are less than other studies have shown and recommended [34,36]. The latter studies reported that in order to keep plasma 25(OH)D levels > 75 nmol/L, it is recommended that the daily allowance of vitamin D be 1000-2000 IU/day and may go up to 6000 IU/day in case of pregnant women [36].

## Conclusions

25(OH) D concentrations are very low among Jordanians that could imply risk of musculoskeletal and metabolic diseases associated with hypovitaminosis D. Thus, it is essential to correlate these low 25(OH) D levels with bone disease parameters and other metabolic diseases among Jordanians. It is important, however, that all Jordanians especially females, should have enough supplements of (1000 U/day) vitamin D [34,36]. Furthermore, fortification of food on a national basis with vitamin D is necessary to overcome such low levels; and determining the association of SNPs markers of inactivating enzymes and/or vitamin D binding protein that may contributed to the low plasma 25(OH)D levels among Jordanians are warranted. If these SNPs do exist then the dose of vitamin D supplementation should be adjusted.

## Competing interests

The authors declare that they have no competing interests.

## Authors' contributions

EM carried out the experimental work, participated in the clinical part and drafted the manuscript. MF participated in the analysis of samples. ME participated in the experimental work and clinical part. NQ participated in the clinical part and revised the manuscript. NI participated in the statistical analysis and revised the manuscript. TA conceived the study, designed the CRF and helped in recruiting volunteers. KM participated in the CRF, sample analysis, statistical analysis and edited the manuscript. All authors read and approved the final version of the manuscript.

## Pre-publication history

The pre-publication history for this paper can be accessed here:

http://www.biomedcentral.com/1472-6890/11/8/prepub
